# Social determinants of health and the double burden of disease in Nepal: a secondary analysis

**DOI:** 10.1186/s12889-022-13905-3

**Published:** 2022-08-17

**Authors:** Hannah Gardner, Georgina Miles, Ayesha Saleem, Aleksandra Dunin-Borkowska, Hannah Mohammad, Natasha Puttick, Sanam Aksha, Suraj Bhattarai, Claire Keene

**Affiliations:** 1 Institute of Human Sciences, University of Oxford, Oxford, UK; 2 Medical Sciences Division, University of Oxford, Oxford, UK; 3 UCL Medical School, University College London, London, UK; 4 Faculty of Medicine, Imperial College London, London, UK; 5 Barts and The London School of Medicine and Dentistry, Queen Mary University of London, London, UK; 6 School of Public Administration, National Center for Integrated Coastal Research, University of Central Florida, Orlando, USA; 7 Department of Global Health, Global Institute for Interdisciplinary Studies, Kathmandu, Nepal; 8 Health Systems Collaborative, Oxford Centre for Global Health Research, University of Oxford, Oxford, UK

**Keywords:** Nepal, Double burden of disease, Social determinants of health, Rural, Urban, Infectious disease, Non-communicable disease, Urban penalty

## Abstract

**Background:**

As the global burden of disease evolves, lower-resource countries like Nepal face a double burden of non-communicable and infectious disease. Rapid adaptation is required for Nepal’s health system to provide life-long, person-centred care while simultaneously improving quality of infectious disease services. Social determinants of health be key in addressing health disparities and could direct policy decisions to promote health and manage the disease burden. Thus, we explore the association of social determinants with the double burden of disease in Nepal.

**Methods:**

This is a retrospective, ecological, cross-sectional analysis of infectious and non-communicable disease outcome data (2017 to 2019) and data on social determinants of health (2011 to 2013) for 753 municipalities in Nepal. Multinomial logistic regression was conducted to evaluate the associations between social determinants and disease burden.

**Results:**

The ‘high-burden’ combined double burden (non-communicable and infectious disease) outcome was associated with more accessible municipalities, (adjOR3.94[95%CI2.94–5.28]), municipalities with higher proportions of vaccine coverage (adjOR12.49[95%CI3.05–51.09]) and malnutrition (adjOR9.19E103[95%CI19.68E42-8.72E164]), lower average number of people per household (adjOR0.32[95%CI0.22–0.47]) and lower indigenous population (adjOR0.20[95%CI0.06–0.65]) compared to the ‘low-burden’ category on multivariable analysis. ‘High-burden’ of non-communicable disease was associated with more accessible municipalities (adjOR1.93[95%CI1.45–2.57]), higher female proportion within the municipality (adjOR1.69E8[95%CI3227.74–8.82E12]), nutritional deficiency (adjOR1.39E17[95%CI11799.83–1.64E30]) and malnutrition (adjOR2.17E131[95%CI4.41E79-1.07E183]) and lower proportions of population under five years (adjOR1.05E-10[95%CI9.95E-18–0.001]), indigenous population (adjOR0.32[95%CI0.11–0.91]), average people per household (adjOR0.44[95%CI0.26–0.73]) and households with no piped water (adjOR0.21[95%CI0.09–0.49]), compared to the ‘low-burden’ category on adjusted analysis. ‘High burden’ of infectious disease was also associated with more accessible municipalities (adjOR4.29[95%CI3.05–6.05]), higher proportions of population under five years (adjOR3.78E9[95%CI9418.25–1.51E15]), vaccine coverage (adjOR25.42[95%CI7.85–82.29]) and malnutrition (adjOR4.29E41[95%CI12408.29–1.48E79]) and lower proportions of households using firewood as fuel (adjOR0.39[95%CI0.20–0.79]) (‘moderate-burden’ category only) compared to ‘low-burden’.

**Conclusions:**

While this study produced imprecise estimates and cannot be interpreted for individual risk, more accessible municipalities were consistently associated with higher disease burden than remote areas. Female sex, lower average number per household, non-indigenous population and poor nutrition were also associated with higher burden of disease and offer targets to direct interventions to reduce the burden of infectious and non-communicable disease and manage the double burden of disease in Nepal.

**Supplementary Information:**

The online version contains supplementary material available at 10.1186/s12889-022-13905-3.

## Background

The global burden of disease has shifted dramatically over the past 30 years [1]. The proportion of global deaths due to non-communicable diseases increased from 55% in 1990 to 71% in 2016, most of which was due to cancers, cardiovascular diseases, chronic respiratory diseases, and diabetes [2]. This shift does not only affect the elderly in affluent societies: 15 million people aged 30 to 69 years die prematurely each year due to non-communicable disease, and 85% of these deaths are in lower and middle-income settings [3]. In many settings, such as Nepal, the epidemiological transition from infectious to non-communicable drivers of morbidity and mortality is ongoing, resulting in a double burden of a concurrent high burden of chronic and infectious disease [4].

Nepal is one of the poorest countries in South Asia with 21.6% of the population living below the national poverty line [5], and has emerged from a decade-long conflict starting in the mid-1990s, followed by another decade of political transition [6]. Despite this, Nepal has made substantial improvements in social, economic and political spheres, as evidenced in the increase in its Universal Health Coverage Index from 48 in 2017 to 53 in 2019 [7], its Human Development Index from 0.387 in 1990 to 0.602 in 2019, and the mean years of schooling increasing from 2 to 5 years over the same period [8]. In 2021, Nepal was recommended to graduate from a ‘least developed country’, which will take effect in 2026 [9].

The epidemiological transition has seen an increase in non-communicable diseases in Nepal, which represented nearly two-thirds of total deaths in 2015, compared to less than 30% in 1990 [10]. This mortality, particularly due to diabetes and cardiovascular diseases, is projected to rise alongside socioeconomic development [11]. Many common causes of non-communicable mortality also result in years lived in disability prior to death, such as diabetes, which is also the 11^th^ most common cause of disability-adjusted life years in Nepal [1]. Furthermore, multimorbidity (defined as occurrence of two or more chronic conditions) was found to be present in 13.96% of participants of a recent nationally representative survey [12]. However infectious disease rates are still high, with 86% of adult mortality in one province attributed to infections [13], representing a double burden of disease [14].

The shifting global disease burden has highlighted the influence of ‘*the conditions in which people are born, grow, live, work and age’* on disease burden, termed ‘social determinants of health’ [15]. The social determinants of health framework has been cited as a “neglected paradigm” in Nepal, due to insufficient awareness or research on the impact of social determinants on disease burdens and health outcomes [16]. Nepal faces ongoing demographic shifts, changing patterns of diet, physical activity, alcohol and tobacco consumption [11]. Key social determinants in Nepal include politics, poverty, education, employment, gender, ethnicity, social capital, housing and sanitation, food security and access to healthcare [13].

This study aimed to quantify the distribution of the double burden of disease in Nepal, and describe associations with social determinants of health, in order to support evidence-informed decision-making to address health inequities.

## Methods

### Study design

This is a retrospective cross sectional, ecological-level, quantitative analysis of publicly available, aggregated, sub-national data.

### Study setting and population

Nepal is a landlocked country characterised by a challenging terrain, ethnolinguistic diversity, and high levels of poverty [6]. It has a population of around 30 million, who belong to over 126 ethnic groups in seven provinces [17]. Topographically, Nepal is divided into three distinct ecological zones: Mountain, Hill, and Tarai. Because of the geological formation of the Himalayan mountains, the country is vulnerable to a multitude of natural hazards such as floods, landslides, and earthquakes [18]. Although trending upwards, unemployment in Nepal is relatively low, reaching 5.1% of the total labour force in 2021 [19]. However, much of the economy is still dependent on agriculture, forming 65.7% of employment although the service sector is the largest contributor to GDP [20].. Consequently, a substantial labour force is out-migrated, which plays a key role in boosting household incomes and Nepal’s economic development [6]. This process has also reduced the available workforce in remote communities and significantly increased the household burden of the women who are already overburdened by household activities.

In 2015 Nepal was affected by earthquakes that killed more than 9,000 people and caused widespread destruction of houses and critical infrastructure, including schools and healthcare facilities [18]. This event invigorated the ongoing constitution-writing process, which affirmed the fundamental right to healthcare in Nepal [21]. The newer constitution adopted a three-tier governance system in Nepal: federal, province, and municipality. Currently, there are seven provinces and 753 *Palikas* (metropolises, sub-metropolises, municipalities, and *gaunpalikas*) [22]. In general, *Gaunpalikas* are considered rural areas, whereas other municipalities are regarded as urban areas. However, based on several indicators including the availability of transport facilities (standard and regularity of road and air transport facilities), distance from district headquarters, distance from the provincial capital, the status of health, human development index, geographical locations, availability of education facilities, access to electricity and telecommunication facilities, these municipalities are further classified into four categories: very remote (162 municipalities), remote (218), fairly accessible (275), and accessible (98) [23, 24]. This study adopted the same classification strategy and grouped municipalities into these four categories to make data comparable and fairly distributed across the spectrum. Municipality and *Palika* are used interchangeably throughout.

### Data Sources

This study was conducted in conjunction with the Global Institute for Interdisciplinary Studies (GIIS), Kathmandu, Nepal, and utilised publicly available, aggregated data from Nepalese governmental sources. This included: the 2011 census data from the Central Bureau of Statistics, 2017–18 and 2018/19 annual health data from the Department of Health Services, and 2019 unemployment data from the Office of the Prime Minister and Council of Ministers. The respective government units received ethical approval from the concerned authorities for primary data collection. Despite its age, the census data is the only source of the scope required to analyse associations at the local level. The dataset includes burden data on disease outcomes from the 2017–2018 and 2018–2019 fiscal as these are the only available datasets at the local level after the adoption of the new constitution.

The data can be disaggregated to either province level (seven provinces), district level (77 districts) or municipality level (753 municipalities). The data is presented as the burden of disease outcomes and of social determinants of health at each *Palika* (municipality) level. Thus, the analysis is at the ecological level for the social determinant and outcome variables. Findings refer to how certain social determinant variables are associated with the burden of disease in a local population, rather than how social determinants of health affect health at the level of the individual.

### Variables

Social determinants of health are ‘*the conditions in which people are born, grow, live, work and age’ *[15]. The 15 social determinant variables, summarised in Table 1, were selected as being most pertinent to the analysis of Nepal from a wider list of 25 variables from the same data sources. The selection was made on the basis of a narrative review [25, 26] of associations between social determinants and disease (aligning with the list of key social determinants of health in Nepal described by Dahal and Subedi in 2015 [27]), discussion of the interpretability of the variables, reduction of overlap between the social determinants, and an initial exploration of the data. The dependent outcome variables included incidence data for the adult population, as new cases presenting to health facilities over 2017–2019, for non-communicable and infectious diseases (termed ‘burden’ in this manuscript). These are presented both as individual diseases and grouped as the infectious, non-communicable or combined double burden of both infectious and non-communicable disease (Table 1).

**Table 1:** List of variables used in this study

*Independent variables:*	*Dependent outcome variables*
***Social Determinants of Health***	**Disease outcomes**
**Demographics**	**Non-communicable disease burden (percentage)**
•Percentage population under 5 years	•Hypertension
•Percentage population over 65 years	•Diabetes
•Percentage female	•COPD
•Percentage of the population absent from place of residence	•Liver cirrhosis
•Percentage indigenous population (a marginalised group in Nepal)	•Depression
•Percentage illiterate	•Back pain
•Percentage population unemployed	**Infectious disease burden (percentage)**
**Household description**	•Tuberculosis
•*Average number of people per household Percent households without a cell phone or landline*	•Malaria
•Percentage households without piped water access	•Leishmaniasis (Kala azar)
•Percentage households that use firewood as a fuel source	•Leprosy
**Access and health**	•Lymphatic filariasis
•Categorisation by degree of accessibility	•HIV
•Percentage of population received two measles, mumps and rubella (MMR) vaccines	•Influenza
•Percentage population malnourished	**Combined outcomes of disease burden**
•Percentage population with nutrient deficiency	•Combined non-communicable disease burden
	•Combined infectious disease burden
	•Combined burden of infectious and non-communicable disease

### Categorisation of the outcome variables

Thresholds to categorise the outcome variables were not readily available in the literature, nor was cluster analysis successful in partitioning data. Therefore, the outcome data were categorised into ‘high’, ‘moderate’ and ‘low’ tertiles. Certain infectious diseases (malaria, leprosy and measles) were absent from more than a third of *Palikas,* making the ‘moderate burden’ tertile threshold zero and meaning that more than one third of *Palikas* were classified as ‘low burden’ and fewer than a third as ‘moderate burden’. In other cases (leishmaniasis, lymphatic filariasis, HIV, Dengue), the ‘high threshold’ identified with this methodology was also zero because more than two thirds of the *Palikas* had zero incidence. In these cases, no moderate category was created, and the data was effectively dichotomised between a ‘low burden’ and a ‘high burden’ category.

Three combined outcome variables were generated to represent the infectious, non-infectious and total disease burden in each *Palika. Palikas* were categorised into low, moderate and high burden for each of these variables according to the relative size of the diseased population, using the criteria detailed in Table 2. These criteria were derived from the median number of individual diseases categorised as high or moderate in a municipality for either infectious diseases or non-communicable diseases. Those with more than the median number of individual diseases categorised as ‘high’ (two for non-communicable and three for infectious diseases) were assigned a combined outcome category of ‘high’. Those with more than the median number of individual diseases categorised at ‘moderate’, but fewer than the median categorised as ‘high’ were assigned a combined outcome category of ‘moderate’. The combined double burden categories were determined by the high, moderate or low statuses of infectious and non-communicable disease burden in that *Palika*, as outlined in Table 2

**Table 2:** C*riteria for categorisation of combined outcome variables for each Palika*

**Burden category for each *Palika***	***Number of individual diseases categorised as high, moderate or low in a municipality***		
	Combined non-communicable disease burden	Combined infectious disease burden	Combined double burden of disease burden
**High burden**	> 2 high, any moderate, any low	> 3 high, any moderate, any low	ID and NCD both high, or one high one moderate
**Moderate burden**	≤ 2 high, > 2 moderate, any low	≤ 3 high, > 1 moderate, any low	ID and NCD both moderate, one high one low, or one moderate and one low
**Low burden**	All others	All others	ID and NCD both low

*ID* Infectious disease. *NCD* Non-communicable disease

### Analysis

The analysis was conducted in SPSS Statistics 27 (2020) [28]. Visualisation of the data confirmed that the distributions of model residuals from the dataset did not meet the assumptions of general linear models of regression. Multinomial logistic analysis was conducted with the reference category as ‘low’. Univariate analyses were conducted. Multivariable regression was conducted for each outcome to adjust for confounding, initially including all 15 variables then using a backwards stepwise approach (removal probability was 0.05). Likelihood odds ratios with 95% confidence intervals are presented. Mapping the distribution of outcomes in Nepal by *Palika* was conducted in SPSS Statistics 27 (2020) [28] using a *Palika-*level shapefile obtained from the Department of Survey, Government of Nepal [29].

## Results

The distribution of social determinants are described in Table 3: overall and for each type of *Palika.* The median proportion of females in each municipality was 52% [IQR 51% – 54%). The median proportion of the population over 65 years was just over 5% (IQR 4% -7%) and the median proportion under 5 years old was just over 10% (IQR [9% -12%). The median proportions of people with no phone access was (42% [IQR 28 – 56%]), no piped water access (35% [IQR 17% – 85%]) and illiteracy (30% [IQR24% – 37%]) were large. However, a median of nearly 70% of the population had received two doses of the measles, mumps and rubella (MMR) vaccine (IQR 58% -83%).

**Table 3:** Description of the overall distribution of social determinants of health for the 753 Palikas (municipalities) in Nepal

***Social Determinants of Health***	*Overall population*	*Palikas*			
		Very remote	Remote	Fairly accessible	Accessible
*Number of municipalities (n)*	753	163	219	273	98
	Median [IQR]	Median [IQR]	Median [IQR]	Median [IQR]	Median [IQR]
*Percentage population under 5 years*	10.187 [8.717, 12.115]	10.522 [9.042, 12.399]	9.471 [8.416, 11.514]	8.693 [7.375, 9.429]	7.234 [6.142, 8.094]
*Percentage population over 65 years*	5.433 [4.278, 7.225]	5.733 [4.524, 7.951]	5.003 [3.952, 6.259]	2.136 [1.170, 3.878]	4.723 [3.136, 5.581]
*Percentage illiterate*	30.148 [23.865, 37.470]	31.536 [26.253, 38.214]	28.120 [21.670, 36.534]	21.528 [17.599, 30.617]	14.509 [11.672, 17.872]
*Percentage female*	52.261 [50.510, 54.030]	52.296 [50.591, 54.229]	52.271 [50.442, 53.855]	50.781 [48.991, 52.889]	49.044 [47.528, 52.114]
*Percentage population unemployed*	2.609 [0.926, 5.435]	3.565 [1.311, 6.535]	1.566 [0.741, 3.646]	0.678 [0.284, 1.537]	0.044 [0.008, 0.088]
*Percent households without a cell phone or landline*	41.834 [27.838, 55.834]	47.398 [35.385, 61.349]	32.108 [18.834, 46.837]	11.285 [5.767, 21.472]	13.142 [2.619, 23.565]
*Percentage households without piped water access*	35.331 [17.238, 85.066]	27.586 [15.110, 72.282]	57.704 [22.768, 90.506]	68.420 [24.879, 79.374]	49.708 [33.338, 69.043]
*Percentage households that use firewood as a fuel source*	92.167 [62.252, 98.409]	96.931 [83.343, 98.790]	76.680 [53.917, 93.206]	48.175 [34.885, 70.239]	23.028 [4.607, 36.641]
*Percentage of the population absent from place of residence*	6.812 [3.649, 10.205]	6.841 [3.569, 10.618]	6.903 [3.780, 9.961]	5.749 [2.698, 8.714]	6.300 [3.097, 9.661]
*Average number of people per household*	4.898 [4.449, 5.590]	4.951 [4.509, 5.660]	4.770 [4.408, 5.473]	4.530 [4.212, 5.302]	4.059 [3.836, 4.491]
*Percentage indigenous population*	30.662 [8.616, 48.426]	35.651 [7.529, 57.454]	27.729 [8.447, 39.789]	36.217 [9.895, 41.03]	21.904 [14.38, 30.662]
*Percentage of population received two MMR vaccines*	69.45 [57.95, 82.55]	66.275 [55.025, 78]	77.275 [62.125, 87.975]	83.7 [75.7, 101.3]	73.925 [40.15, 76]
*Percentage population malnourished*	0.356 [0.098, 0.866]	0.341 [0.073, 0.915]	0.407 [0.159, 0.826]	0.253 [0.159, 0.354]	0.304 [0.189, 0.797]
*Percentage population with nutrient deficiency*	0.057 [0.016, 0.162]	0.049 [0.011, 0.139]	0.076 [0.024, 0.181]	0.024 [0.008, 0.079]	0.13 [0.071, 0.238]

The distribution of disease burden is described in Table 4. Back pain (a median of 2.862% [IQR 1.855—4.607]) and hypertension (median of 1.562% [IQR 0.792—2.907]) had the highest burden over the two-year period, and pneumonia had the highest burden among the infectious diseases (a median of 1.174% [0.609—2.020]).

**Table 4:** Distribution of non-communicable and infectious disease incidence over two years as a percentage of population for all Palikas in Nepal

	*Median [IQR]*	*Minimum*	*Maximum*
***Non-communicable disease incidence (percentage)***			
* Hypertension*	1.562 [0.792, 2.907]	0.017	55.987
* Diabetes*	0.141 [0.018, 0.538]	0.000	33.054
* COPD*	0.909 [0.438, 1.788]	0.000	39.500
* Liver cirrhosis*	0.023 [0.005, 0.584]	0.000	4.543
* Depression*	0.017 [0.000, 0.077]	0.000	12.036
* Back pain*	2.862 [1.855, 4.607]	0.159	76.229
***Infectious disease incidence (percentage)***			
* Tuberculosis*	0.133 [0.0791, 0.209]	0.000	0.526
* Malaria*	0.000 [0.000, 0.006]	0.000	0.872
* Leishmaniasis (Kala azar)*	0.000 [0.000, 0.000]	0.000	0.221
* Leprosy*	0.004 [0.000, 0.017]	0.000	9.752
* Lymphatic filariasis*	0.000 [0.000, 0.000]	0.000	0.369
* HIV*	0.000 [0.000, 0.000]	0.000	0.588
* Influenza*	0.808 [0.185, 2.247]	0.000	17.583
* Pneumonia*	1.174 [0.609, 2.020]	0.0398	10.807
* Measles*	0.000 [0.000, 0.005]	0.000	0.190
* Dengue*	0.000 [0.000, 0.000]	0.000	0.117

**Fig. 1:**
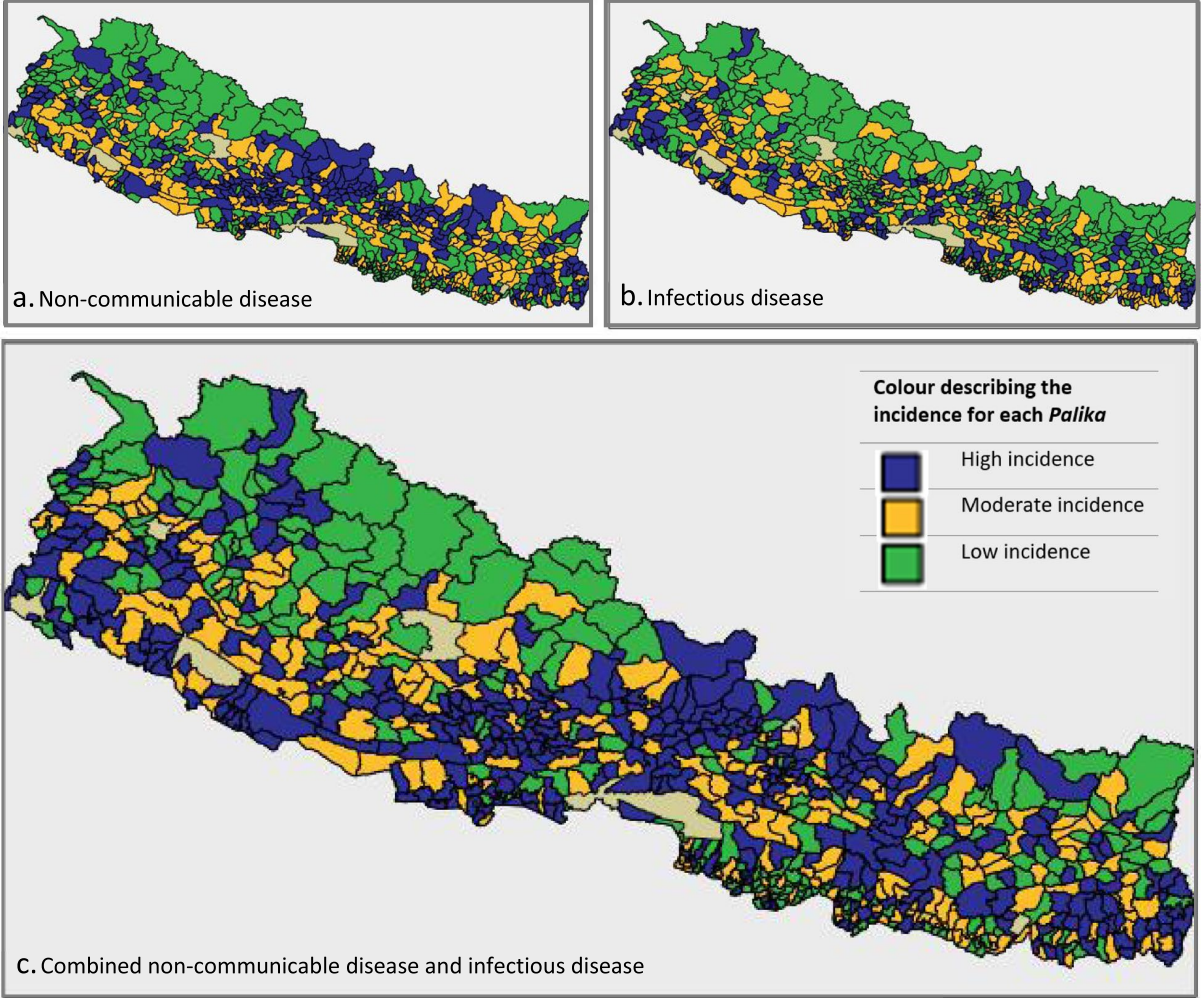
Distribution of the double burden of infectious and non-communicable disease in Nepal for 2017–2019

A high burden of non-communicable disease was distributed across the central parts of Nepal, with municipalities with high burden of infectious disease distributed more sparsely (Fig. 1a and b). Of the 753 municipalities, 189 were classified as having a high double burden of disease burden, 413 were classified as moderate and 151 were classified as low double burden of disease burden (Fig. 1c).

*This figure was created in SPSS Statistics 27 2020* ^*28*^ *.*

**Fig. 2:**
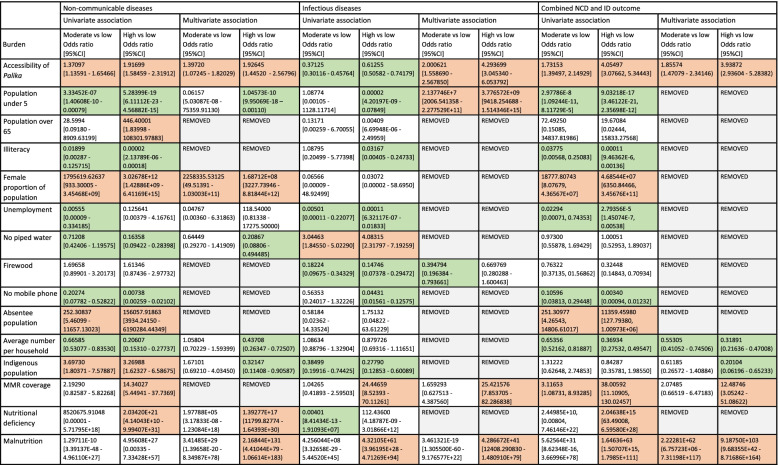
Associations of social determinants of health with combined disease outcomes. Red: significant (*p* ≤ 0.05) positive association (social determinant of health associated with more disease). Green: significant (*p* > 0.05) negative association (social determinant of health associated with less disease). Removed: variable removed by model in backwards selection multivariable analysis

The univariate and adjusted associations of the social determinants of health and the combined non-communicable disease, infectious disease and combined double burden outcomes are described in Fig. 2. More accessible *Palikas* were associated with higher burden of combined infectious, non-communicable and the double burden of disease for all adjusted analyses.

The combined non-communicable outcome was also associated with higher proportions of women in a municipality, nutritional deficiency, and malnutrition (the latter two only high vs low burden) and lower proportions of population under five years old, indigenous population, lower number of average people per household and lower proportions of no piped water in the municipality (all high vs low only) on adjusted analysis. The combined infectious disease outcome was associated with higher proportions of the population under five years old, MMR coverage and malnutrition (latter two high vs low only) and lower proportions of the municipalities using firewood as fuel (moderate vs low only) on adjusted analysis.

The combined double burden outcome was associated with a higher proportion of MMR coverage (high vs low only) and malnutrition and lower average number of people per household and indigenous population (high vs low only) on adjusted multivariable analysis.

The univariate and adjusted multivariable associations between the social determinants of health and the individual disease outcomes can be found in the Supplementary material (S1-4).

## Discussion

This study evaluated the distribution of disease outcomes in Nepal and the association of municipal-level prevalence of social determinants of health with infectious and non-communicable disease burden. Our study found a wide distribution of both infectious and non-communicable disease, with more than half the *Palikas* considered as having a moderate or high double burden of both. This finding reflects the rising burden of non-communicable disease against a background of high infectious disease burden. Thus, Nepal is increasingly bearing a “double burden” of disease, associated with a number of social determinants of health.

### Urbanisation as a driver of disease

Our study reported higher burden of infectious, non-communicable and a double burden of disease in populations in more accessible than remote *Palikas* on adjusted analysis. The literature suggests that urban households have health advantages compared to remote settings, as they typically spend more on housing, food, education and healthcare [30]. Our findings counteract this notion of the ‘urban advantage’ and highlight how disease outcomes cannot be assumed to improve with economic growth and demographic change [31]. Within accessible areas, the impact of socioeconomic inequalities continues to grow with households in poor neighbourhoods and slums frequently experiencing worse health outcomes [30]. For example, in Kathmandu rapid levels of migration from very remote to more accessible municipalities, at a rate of 4% per year, has led to the creation of multiple temporary settlements [32]. These communities often settle on undeveloped land on the banks of rivers, subjecting residents to multiple health risks ranging from poor housing and sanitation to inadequate sewage, drainage and drinking facilities that increases vulnerability to infectious diseases [33].

Behavioural risk factors associated with non-communicable disease, such as tobacco consumption, alcohol use, physical inactivity and poor diets, are generally more prevalent in urban environments as well [34]. While the burden of non-communicable disease was higher in accessible than very remote municipalities in this study, the burden has been reported to be rising in across all regions [35]. Increasing rates of non-communicable disease in remote areas may be associated with migration to more accessible *Palikas,* which facilitates the transference of urban influences, such as ‘junk’ food and low physical activity-based lifestyles, to remote areas [34].

The rapid and often uncontrolled rate of population growth in Nepal has also been associated with increased risk of air and water pollution [36], linked to both non-communicable health problems (such as chronic obstructive pulmonary disease [36]) and infectious diseases (such as water-borne pathogens, reported as the third leading cause of inpatient morbidity in Kathmandu [37]). The quality and availability of drinking water has long been an area of concern, with nearly 50% of Kathmandu’s population surviving on groundwater which has been found to be contaminated with industrial and domestic waste [38], and demand for drinking water increasing at a rate of 5% annually [37]. Thus, Nepal’s accessible *Palikas* are increasingly susceptible to the double burden of disease.

The association of the social determinants of health with disease outcomes may be confounded by their unequal distribution across accessible, fairly accessible, remote and very remote municipalities. These include variables such as piped water and firewood (both more prevalent in more remote areas) and vaccine coverage, which is better in more accessible areas. Rapid urbanisation has seen the number of urban centres increasing from 58 in 2013 to 293 in 2017 [39]. Several independent factors have been cited as triggering Nepal’s urbanisation, including the demographic transition of more people entering the labour force than leaving; a geographic transition with migration from remote to more accessible *Palikas*; and an economic transition due to a decline in agriculture [32]. Thus, urbanisation is likely to continue to rise, and its effect on health in Nepal should be considered in further policy.

### Association of demographics and household descriptions with disease outcomes

Our study reported an association between a higher proportion of the population aged under five years and reduced burden of non-communicable diseases, in particular hypertension and back pain. This is unsurprising given both tend to be diseases of older age. For infectious diseases however, a greater proportion of the population under five was associated with higher burden of infectious diseases in the adult population, in particular for pneumonia, tuberculosis and leprosy, reflecting the impact that the age structure of a population has on the disease trends. Childhood mortality rates in Nepal have reduced by over 50% in the last 15 years, largely characterised by reduced prevalence of infectious disease [40], but higher proportions of children still seem to present a risk factor for adult infections. This may, however, be confounded by higher proportions under five in more remote municipalities, possibly reflecting rural–urban economic migration of working-age people. Nepal also has a relatively large population of younger people, characteristic of a low-middle income nation [41], which could continue to drive the high infectious disease burden. This makes efforts to reduce infectious disease-related childhood deaths critical in improving overall health outcomes.

Municipalities with higher proportions of females did not have higher infectious disease incidence overall and they were associated with lower incidence of tuberculosis, Kala azar and filariasis on adjusted analysis. Conversely, a higher female proportion was strongly associated with non-communicable disease incidence, with particularly strong associations for hypertension, COPD (Chronic Obstructive Pulmonary Disease) and liver cirrhosis. Whilst both men and women are affected by non-communicable diseases, the risk factors diverge significantly: globally, women are more likely to have biological risk factors, such as higher body mass or raised blood pressure [42], and this pattern is also demonstrated in Nepal [43]. While men tend to participate in risky behaviours such as tobacco smoking, Nepalese women are reported to smoke at a rate far above the global average for smoking amongst women [44–46]. Research on Nepalese women has also demonstrated that risk factors were clustered around more vulnerable groups such as widowed or separated women, including Dalit women [47] (although our study found municipalities with a higher proportion of indigenous population to be associated with lower incidence of non-communicable disease overall).

In addition to biological risk, the proportion of females may reflect an associated socioeconomic risk. Women’s roles have changed with the out-migration of the male population; of the 4 million Nepalese working outside the country, 90% are male [48]. Thus a high proportion of women may represent an underlying economic vulnerability in the need for economic migration to support families but also reflect a situation of shifting roles, with female headed households meaning women take on more responsibility for work and decision-making [48]. However, despite their importance women have highly restricted economic ownership of natural resources, thus limiting their social development [49]. Nepal has a deep running caste system, with those at the lowest level (Dalit) experiencing increased vulnerability to a range of undesirable outcomes, chiefly poverty [50]. Notably, lower caste women are more vulnerable than both lower caste men and upper caste women [51]. The exposure of such women to alcoholism, illegal trafficking, and drug abuse in post-disaster contexts demonstrates their vulnerability to external insult and the fragility of their health status [52].

Higher proportions of indigenous populations in a municipality were associated with lower burden of non-communicable disease and the double burden of disease, but not with infectious disease overall. Indigenous populations in Nepal exist within a somewhat liminal state. The Nepalese population is composed of 126 different caste and ethnic groups, with indigenous people representing nearly half the population [53]. Indigenous communities face a number of challenges including discrimination in healthcare services [53]. The lower burden of non-communicable disease associated with larger indigenous populations may reflect either the lower association with diseases of affluence that non-communicable diseases tend to represent, or this finding may be confounded by poorer access to healthcare and thus lower reported burden. It could also represent the complex interaction of social determinants of health and the differing impact they have on outcomes depending on context [54].

We also observed an association between a higher average number of people per household and a reduced burden of non-communicable and the double burden of disease on adjusted analysis. The median average number of people per household was similar across remote and accessible municipalities. Nonetheless, multigenerational households may be associated with poverty and a subsequent association with reduced risk of ‘diseases of lifestyle’ [45]. Average household size was found to be unimportant in infectious disease burden in contrast to research on the environmental determinants of disease prevalence in rural western Nepal, which found an association between reduced burden of infectious disease and larger average household size [55].

### Health and healthcare access

Malnutrition was associated with a higher burden of infectious, non-communicable and the double burden of disease on adjusted analysis. Specific nutrient deficiency was also associated with higher non-communicable disease rates. In accordance with the ongoing demographic transition, Nepal has experienced a shift from an agricultural-based diet to a highly processed one [56]. There are well-documented associations between malnutrition and nutrient deficiency and non-communicable diseases such as diabetes [57]. Poor nutrition status may also be indicative of communities with poor access to resources [58], due to factors such as geographical isolation or poverty [59]. Malnutrition, particularly in childhood, is becoming increasingly prevalent in urban areas, which are linked to increased rates of disease both in this study and the literature [60]. In this study, nutrient deficiency was associated more with accessible *Palikas*, but malnutrition was not—possibly reflecting poor quality nutrition as well as obesity in urban areas more than a lack of nourishment altogether [61, 62].

Vaccination rates across Nepal vary and the percentage of children who completed the MMR vaccination schedule can be used as a proxy of healthcare access, with greater MMR coverage reflective of greater healthcare access. The median vaccination rate was 70% across the 753 municipalities, echoing literature that cite less than 80% of children as fully immunised [63]. The vaccination coverage was similar across remote and accessible municipalities, supporting previous evidence that highlighted how geographical location and the urban/rural divide do not contribute to differences in immunisation coverage [64]. Higher vaccination coverage was associated with a higher incidence of infectious disease overall and individually with diabetes, COPD and back pain on adjusted analysis. Increased rates of disease in areas with greater vaccination coverage may be explained by higher rates of testing and health reporting if seen as a proxy of healthcare access [65].

### Limitations

There are several important limitations that restrict the inferences that can be drawn from our findings. The first to consider is the ecological fallacy. Inferences on individual risk cannot be drawn from the municipality level data. The disease outcome and social determinant data were also collected at different time points, so there may be changes in the social determinant prevalence that was unaccounted for at the time the disease burden data was collected. However, this data is the best quality available in Nepal and the findings can still provide an incremental step towards a greater understanding of the dynamics of disease burden in Nepal. Interventions to address disease burden must respond to the realities of the population dynamics and the impact on disease [66], thus even imperfect information is better than no information and could inform broad decisions.

The interpretation of variables was not always clear: ‘no recorded disease’ in a municipality may have reflected less accurate health information systems rather than zero burden, and there might be differences in reporting of disease across municipalities. Wealthier and urban municipalities may have more robust reporting, particularly with regards to self-reporting, and therefore this could bias the results. The social determinants of health were interpreted as proxies for socioeconomic status and the rural–urban distribution of municipalities. For example, ‘no piped water’ in a community may be seen to reflect the conditions of poverty, ruralness and water and sanitation prospects in an area, rather than that there is simply no piped water. Government implementation of territorial reform following the 2015 constitution also incentivised remote administrative units to amalgamate into larger entities or be absorbed into ‘accessible’ *Palikas*, so may skew the categorisation of very remote to accessible municipalities and confound the associations.

The epidemiological transition within Nepal has affected patterns of morbidity and mortality in addition to incidence. Although due to the limitations of our data this research focused on only incidence rate, the morbidity and mortality burdens are also worth considering in future research.

From a statistical perspective, multinomial regression loses information when continuous variables are categorised [67]. Calculation of disability adjusted life years could also improve the specificity of the findings in future research. Additionally, the categorisation of outcomes by tertiles into ‘high’, ‘moderate’ and ‘low’ burden is somewhat artificial and prevents absolute comparisons to other settings as the categorisation is relative within the range of Nepalese municipalities. However, this approach brought some consistency to this particular analysis as it allowed for a comparative analysis within the dataset and thus, stronger conclusions to be drawn.

## Conclusion

This study demonstrated that Nepal is afflicted by a double burden of both high incidence of infectious and non-communicable disease. However, the country is not uniformly impacted, with some areas more affected by infectious disease, some by non-communicable disease, and some areas experience a double burden of both. While this study produced imprecise estimates and cannot be interpreted for individual risk, more accessible municipalities were consistently associated with higher disease burden than smaller, more remote areas. Social determinants that appear to represent lower socioeconomic status had mixed associations with health outcomes, but female sex, lower average number of people per household, non-indigenous population and poor nutrition were associated with higher burden of disease. These identified social determinants offer targets to direct interventions in order to manage the double burden of disease in Nepal, and demonstrate the value of country-level data for guiding priorities.

## Supplementary Information

**Additional file 1. **Supplementary Tables.

## Data Availability

The datasets analysed during the current study are available from the corresponding author on reasonable request.

## Abbreviations

MMR: Measles, Mumps and Rubella
COPD: Chronic Obstructive Pulmonary Disease
HIV: Human Immunodeficiency Virus
