# Supplementary material for: Social determinants of health and the double burden of disease in Nepal: a secondary analysis
Source: BMC Public Health. 2022 Aug 17;22:1567. doi: 10.1186/s12889-022-13905-3 (PMC9387078; doi:10.1186/s12889-022-13905-3)
Supplement: Supplementary file 1 — Additional file 1. Supplementary Tables. [file 12889_2022_13905_MOESM1_ESM.docx]

Supplementary Table 1. Univariate associations of social determinants of health with non-communicable disease outcomes (odds ratio [95% CI])

|  | Non-communicable diseases | | | | | | | | | | | |
| --- | --- | --- | --- | --- | --- | --- | --- | --- | --- | --- | --- | --- |
|  | Hypertension | | Diabetes | | COPD | | Liver Cirrhosis | | Depression | | Back pain | |
|  | Moderate vs low | High vs low | Moderate vs low | High vs low | Moderate vs low | High vs low | Moderate vs low | High vs low | Moderate vs low | High vs low | Moderate vs low | High vs low |
| Accessibility | ***1.64081 (1.35433 - 1.98789)** | ***2.20160 (1.80238 - 2.68924)** | ***1.97005 (1.59687 - 2.43044)** | ***4.78256 (3.71595 - 6.15532)** | 1.04805 (0.87444 - 1.25613) | 1.11264 (0.92810 - 1.33387) | 1.06608 (0.88947 - 1.27776) | 1.02591 (0.85605 - 1.22949) | ***1.21562 (1.00987 - 1.46329)** | ***1.85980 (1.53281 - 2.25656)** | 1.09844 (0.91634 - 1.31674) | 1.04804 (0.87445 - 1.25609) |
| Population under 5 | ****4.20069E-18 (3.80497E-22 - 4.63756E-14)** | ****2.98253E-33 (2.27880E-38 - 3.90359E-28)** | ****4.52059E-11 (1.22539E-14 - 1.66769E-07)** | ****1.27180E-27 (2.80342E-32 - 5.76968E-23)** | 0.00590 (3.44581E-06 - 10.08905) | 0.00095 (5.24652E-07 - 1.71603) | 3.90851 (0.00218 - 6991.59480) | 4.78899 (0.00269 - 8531.15227) | 0.00123 (7.44159E-07 - 2.01762) | ****8.86387E-12 (2.42871E-15 - 3.23498E-08)** | 0.13583 (0.00009 - 214.37248) | ****1.40307E-06 (6.12825E-10 - 0.00321)** |
| Population over 65 | ***1.16407E+08 (112598.10830 - 1.20344E+11)** | ***5.49107E+08 (527823.63914 - 5.71249E+11)** | 16.59842 (0.16683 - 1651.45565) | 10.97440 (0.10338 - 1164.98274) | 0.02334 (0.00012 - 4.62921) | 2.03319 (0.08055 - 51.32373) | 0.21072 (0.00229 - 19.41069) | 2.64655 (0.08949 - 78.26527) | 1.94567 (0.04301 - 88.01234) | 2.18580 (0.05034 - 94.90512) | 146.27099 (0.51263 - 41736.04666) | ***399.70105 (1.45157 - 110060.89535)** |
| Illiteracy | ****0.03359 (0.00512 - 0.22028)** | ****1.28724E-06 (1.05764E-07 - 0.00002)** | ****0.04010 (0.00614 - 0.26180)** | ****1.30484E-06 (1.06912E-07 - 0.00002)** | ****0.00370 (0.00055 - 0.02485)** | ****0.00079 (0.00011 - 0.00572)** | 2.48472 (0.41415 - 14.90731) | 0.74724 (0.12152 - 4.59507) | ****0.02078 (0.00328 - 0.13184)** | ****0.00013 (0.00002 - 0.00106)** | ****0.09944 (0.01635 - 0.60496)** | ****0.08944 (0.01465 - 0.54615)** |
| Female proportion of population | ***9.85586E+07 (40648.67042 - 2.38970E+11)** | ***1.99477E+16 (4.94437E+12 - 8.04774E+19)** | ***14637.02847 (9.89858 - 2.16438E+07)** | ***6.24356E+08 (369684.87023 - 1.05447E+12)** | ***7.75325E+09 (3.25829E+06 - 1.84492E+13)** | ***4.05807E+13 (1.37597E+10 - 1.19683E+17)** | ***3874.70149 (3.17482 - 4.72887E+06)** | 16.36052 (0.01347 - 19868.22878) | ***11741.91170 (7.85804 - 1.75454E+07)** | ***1.31099E+09 (756106.77884 - 2.27309E+12)** | ***5110.32639 (3.66920 - 7.11747E+06)** | ***2.62788E+07 (17331.40495 - 3.98453E+10)** |
| Unemployment | ****0.00030 (4.85694E-06 - 0.01821)** | ****0.00078 (0.00001 - 0.04114)** | ****0.00240 (0.00006 - 0.10050)** | ****1.22248E-06 (9.32596E-09 - 0.00016)** | ***5670.82279 (47.54608 - 676359.30415)** | ***61517.32398 (555.26154 - 6.81549E+06)** | ****0.01605 (0.00030 - 0.85067)** | 0.43873 (0.01307 - 14.73047) | 0.45537 (0.01194 - 17.36799) | 0.20084 (0.00474 - 8.51810) | 1.03522 (0.01801 - 59.50749) | 31.04417 (0.74340 - 1296.38699) |
| No piped water | 0.74837 (0.44724 - 1.25223) | **0.**13063 (0.07357 - 0.23194)** | 1.06512 (0.63424 - 1.78873) | 0.80499 (0.47699 - 1.35854) | ****0.29630 (0.17487 - 0.50206)** | ****0.07957 (0.04438 - 0.14267)** | 1.21062 (0.71981 - 2.03609) | 0.97178 (0.57583 - 1.63999) | 0.64152 (0.38292 - 1.07477) | ****0.30983 (0.18128 - 0.52953)** | ****0.46402 (0.27620 - 0.77956)** | ****0.17681 (0.10187 - 0.30687)** |
| Firewood | 0.53564 (0.28154 - 1.01905) | 0.60002 (0.31387 - 1.14706) | 0.78476 (0.40618 - 1.51617) | ****0.44945 (0.23833 - 0.84757)** | ***3.34425 (1.79043 - 6.24657)** | ***4.85618 (2.52393 - 9.34354)** | 1.11428 (0.58742 - 2.11367) | 0.80262 (0.43033 - 1.49699) | ***2.46737 (1.31242 - 4.63872)** | ***2.21047 (1.18466 - 4.12452)** | ***2.04788 (1.09494 - 3.83017)** | ***2.05383 (1.09791 - 3.84203)** |
| No mobile phone | ****0.02547 (0.00874 - 0.07417)** | ****0.00040 (0.00011 - 0.00144)** | ****0.02856 (0.00942 - 0.08662)** | ****0.00005 (0.00001 - 0.00021)** | 0.53097 (0.21626 - 1.30367) | ****0.25498 (0.10275 - 0.63276)** | 0.87550 (0.35794 - 2.14142) | 0.49235 (0.20025 - 1.21049) | ****0.32338 (0.12902 - 0.81052)** | ****0.03763 (0.01421 - 0.09962)** | 0.82870 (0.33846 - 2.02907) | ****0.32047 (0.12949 - 0.79315)** |
| Absentee population | ***36195.35435 (542.91225 - 2.41310E+06)** | ***1.09307E+08 (1.56419E+06 - 7.63852E+09)** | ***326.27747 (7.43203 - 14324.08810)** | ***52722.86442 (1270.39244 - 2.18806E+06)** | ***7964.88111 (176.29314 - 359851.39647)** | ***38827.14193 (868.91106 - 1.73498E+06)** | 26.36639 (0.84497 - 822.73751) | 8.66979 (0.26849 - 279.95264) | ***60.79242 (1.61591 - 2287.07775)** | ***1705.29913 (48.39640 - 60088.05196)** | 26.31018 (0.71472 - 968.53114) | ***971.90014 (28.58956 - 33039.68121)** |
| Average number per household | ****0.44454 (0.34599 - 0.57116)** | ****0.08833 (0.06068 - 0.12860)** | ****0.67537 (0.54145 - 0.84241)** | ****0.26572 (0.20123 - 0.35090)** | ****0.57040 (0.45537 - 0.71449)** | ****0.33370 (0.25794 - 0.43170)** | 1.09162 (0.87979 - 1.35446) | 0.93656 (0.75171 - 1.16686) | ****0.74116 (0.59697 - 0.92018)** | ****0.37725 (0.29293 - 0.48585)** | 0.85083 (0.68732 - 1.05325) | ****0.57799 (0.45911 - 0.72765)** |
| Indigenous population | ***13.45064 (6.07497 - 29.78119)** | ***23.97306 (10.75423 - 53.44011)** | ***2.23901 (1.09381 - 4.58320)** | ***3.91306 (1.92076 - 7.97187)** | 1.39236 (0.70077 - 2.76647) | 0.73955 (0.36701 - 1.49025) | 1.13068 (0.57106 - 2.23870) | 0.50949 (0.25205 - 1.02985) | 1.56157 (0.78205 - 3.11806) | 1.09382 (0.54411 - 2.19892) | 1.01917 (0.50887 - 2.04122) | 1.22741 (0.61524 - 2.44869) |
| MMR coverage | 1.89071 (0.73150 - 4.88696) | ***5.26212 (2.03173 - 13.62874)** | 1.02066 (0.38578 - 2.70038) | ***13.44214 (5.01284 - 36.04565)** | ***11.39799 (4.12153 - 31.52088)** | ***43.19461 (15.16531 - 123.02910)** | 2.23524 (0.86748 - 5.75957) | ***4.22738 (1.63905 - 10.90308)** | ***2.64602 (1.00799 - 6.94592)** | ***11.01776 (4.14721 - 29.27049)** | ***2.98614 (1.14980 - 7.75527)** | ***5.99659 (2.30057 - 15.63050)** |
| Nutritional deficiency | 3.47990E+11 (0.20091 - 6.02737E+23) | ***1.28063E+26 (3.62107E+14 - 4.52909E+37)** | 0.00731 (4.48534E-14 - 1.19121E+09) | ***8.76163E+11 (79.53901 - 9.65138E+21)** | 29.59644 (1.92126E-09 - 4.55925E+11) | 180.28677 (1.38699E-08 - 2.34345E+12) | 26.34405 (1.17736E-09 - 5.89460E+11) | 25584.89991 (2.13345E-06 - 3.06820E+14) | 88.11926 (5.17833E-09 - 1.49952E+12) | 1083.16819 (8.01909E-08 - 1.46308E+13) | 6.15254E+09 (0.00080 - 4.76086E+22) | ***1.92846E+31 (2.22203E+19 - 1.67367E+43)** |
| Malnutrition | 1.19976E-17 (3.37270E-48 - 4.26788E+13) | 2.59484E-19 (2.87827E-50 - 2.33933E+12) | 0.00115 (2.46703E-37 - 5.36941E+30) | 7.46992E+15 (3.07846E-15 - 1.81259E+46) | 1.23218E+07 (9.32888E-38 - 1.62750E+51) | ***2.70673E+65 (1.85977E+27 - 3.93940E+103)** | 6.18819E+22 (3.67289E-26 - 1.04260E+71) | ***3.74324E+88 (1.96853E+45 - 7.11793E+131)** | 7.44142E+24 (3.34038E-14 - 1.65774E+63) | ***1.29092E+47 (9.67633E+10 - 1.72221E+83)** | 1.92846E+31 (2.22203E+19 - 1.67367E+43) | 5.74614E+20 (7.21751E-10 - 4.57472E+50) |

**Bold text with *** indicates significant (p≤0.05) positive association (social determinant of health associated with more disease).

**Bold text with **** indicates significant (p>0.05) negative association (social determinant of health associated with less disease).

Supplementary Table 2. Univariate associations of social determinants of health with infectious disease outcomes (odds ratio [95% CI])

|  | Infectious diseases | | | | | | | | | | | | | | | | | | | |  |
| --- | --- | --- | --- | --- | --- | --- | --- | --- | --- | --- | --- | --- | --- | --- | --- | --- | --- | --- | --- | --- | --- |
|  | TB | | Malaria | | Kala azar | | Leprosy | | Filariasis | | HIV | | Influenza | | Pneumonia | | Measles | | Dengue | |  |
|  | Moderate vs low | High vs low | Moderate vs low | High vs low | Moderate vs low | High vs low | Moderate vs low | High vs low | Moderate vs low | High vs low | Moderate vs low | High vs low | Moderate vs low | High vs low | Moderate vs low | High vs low | Moderate vs low | High vs low | Moderate vs low | High vs low |  |
| Accessibility | | ***2.54413 [2.04336, 3.16764]** | ***4.74109 [3.69260, 6.08729]** | ***5.71682 [3.14892, 10.3788]** | ***1.96227 [1.64399, 2.34217]** | n/a | ***2.86616 [2.03886, 4.02917]** | ***1.81109 [1.47692, 2.22088]** | ***2.07707 [1.72249, 2.50465]** | n/a | ***1.55464 [1.27659, 1.89323]** | n/a | ***3.73421 [2.73466, 5.09909]** | ***1.76201 [1.45540, 2.13321]** | ***1.24092 [1.03141, 1.49301]** | 0.856236 [0.711530, 1.030372] | ****0.578529 [0.478249, 0.699837]** | ***10.3707 [3.97133, 27.0819]** | ***1.53577 [1.30034, 1.81383]** | n/a | ***5.83924 [3.49292, 9.76167]** |
| Population under 5 | | 1.27489E-02 [7.96098E-6, 20.4164] | ****2.89899E-07 [1.24642E-10, 6.74217E+04]** | ****1.44415E-11 [2.70776E-20, 7.70215E-03]** | 34.8827 [5.21562E-02, 2.33299E+03] | n/a | ****1.71027E+06 [8.43052E-12, 0.346954]** | ***8.46961E+03 [2.84301, 2.52319E+07]** | ***1.42862E+05 [1.14325E+05, 1.78522E+08]** | n/a | 6.19486E-03 [2.22119E-6, 17.2773] | n/a | 5.21715E-12 [7.93066E-17, 3.43207E-07] | ****7.13953E-05 [4.00007E-08, 0.127430]** | ****7.28755E-06 [3.75963E-09, 1.41300E-02]** | 1.27828 [5.80323E-04, 2.81566E+03] | ***7.26941E+04 [38.7413, 1.36403E+08]** | ***2.47308E-25 [2.54248E-38, 2.40557E-12]** | ***2.45809E-13 [1.05281E-16, 5.73909E-10]** | n/a | ****8.64405E-21 [7.67632E-29, 9.73378E-13]** |
| Population over 65 | | 0.575062 [2.21432E-02, 14.9344] | **5.57053E-02 [5.52288E-04, 5.5580]** | ****2.03395E-12 [4.71243E-20, 8.7789E-5]** | 4.99829E-02 [5.42431E-04, 4.60573] | n/a | ****6.52061E-14 [2.83993E-19, 1.49716E-8]** | 0.297462 [6.52818E-03, 13.5541] | ****6.95419E-5 [1.68960E-7, 2.862252E-02]** | n/a | 1.04280E-02 [1.71551E-5, 6.338873] | n/a | ****1.21842E-05 [1.56844E-09, 9.46507E-02]** | 1.62000E-03 [1.98950E-06, 1.31671] | ***6.16856E+02 [2.00613, 1.89674E+05]** | 2.29478 [3.04600E-02, 1.72867E+02] | 7.37298 [0.133975, 4.05753E+02] | ***1.03265E-13 [2.22425E-23, 4.79428E-04]** | 1.17944 [5.36000E-02, 25.9510] | n/a | 2.81391 [1.70751E-02, 4.63721E+02 |
| Illiteracy | | 1.69731 [0.288900, 9.97190] | ****3.78135E-02 [5.75209E-03, 0.248582]** | 5.08322E-02 [6.00984E-04, 4.299484] | 3.40195 [0.7125395, 16.2422] | n/a | ****6.42825E-03 [2.87298E-04, 0.143831]** | 1.43173 [0.185314, 11.0616] | ***3.39840E+02 [56.6129, 2.04002E+03]** | n/a | 1.12736 [0.174941, 7.26500] | n/a | ****5.96849E-05 [2.93396E-06, 1.21000E-03]** | ****3.71800E-02 [6.04000E-03, 0.228967]** | ****1.77000E-03 [2.49172E-04, 1.25400E-02]** | ****4.87300E-02 [7.94000E-03, 0.298965]** | ****2.11100E-02 [3.33000E-03, 0.133941]** | ***3.24507E-04 [6.05007E-07, 0.174050]** | ***6.92600E-02 [1.35500E-02, 3.54100E-01]** | n/a | ****8.24052E-7 [6.22864E-9, 1.09023E-04]** |
| Female proportion of population | | 2.49930 [2.18388E-03. 2.86027E+03] | **0.582409 [5.04498E-04. 6.72352E+02]** | ****3.12301E-10 [1.40869E-17, 6.92359E-03]** | ****3.97062E-8 [5.74448E-11, 2.74415E-05]** | n/a | ****8.12215E-09 [1.00365E-13, 6.57296E-04]** | ****9.09059E-6 [3.95039E-9, 2.09192E-02]** | ****1.56093E-13 [8.66258E-17, 2.81269E-10]** | n/a | 2.20932E-05 [1.19323E-8, 4.09069E-02] | n/a | 1.19269 [1.83244E-04, 7.76291E+03] | 66.0024 [4.20651E-02, 1.03561E+05] | 4.26695E+10 [2.12611E+07, 8.56344E+13] | ***2.66362E+06 [1.65666E+03, 4.282628E+09]** | ***8.43462E+08 [4.79904E+05, 1.48244E+12]** | 2.03149E-08 [1.18906E-17, 34.7079] | 1.09907E+02 [0.234400, 5.15329E+04] | n/a | 0.286294 [1.00473E-6, 8.15781E+04] |
| Unemployment | | ****7.92121E-04 [1.67553E-05, 3.74482E-02]** | ****8.19221E-07 [6.16993E-9, 1.08773E-04]** | ****2.69294E-19 [2.59852E-30, 2.79080E-8]** | ****8.52190E-04 [1.55116E-05, 4.68183E-03]** | n/a | ****1.31335E-05 [1.58749E-9, 0.108655]** | ****3.74000E-03 [5.57684E-05, 0.250483]** | ****2.81188E-6 [2.38646E-8, 3.31313E-04]** | n/a | ****2.49230E-04 [1.16189E-6, 5.34607E-02]** | n/a | ****1.42352E-04 [1.64068E-07, 0.123511]** | 0.153914 [3.71800E-02, 9.12715] | 12.760728 [0.346503, 4.699415E+02] | ***5.19637E+08 [40.1934, 1.33607E+06]** | ***3.30113E+06 [2.09714E+11, 5.19637E+08]** | ***1.84394E-28 [3.06700E-47, 1.10862E-09]** | ***3.60000E-03 [7.87167E-05, 0.164241]** | n/a | ****5.39321E-17 [2.90909E-25, 9.99855E-09]** |
| No piped water | | ***5.09432 [2.86298, 9.06471]** | ***9.65826 [5.41059,17.2406]** | ***25.774133 [6.936154, 95.774395]** | ***11.0054 [6.71467, 18.0378]** | n/a | ***2.604398 [1.202061, 5.642714]** | ***32.06957 [15.1359, 67.9481]** | ***2.90453E+02 [1.33687E+02, 6.31049E+02]** | n/a | ***2.49893 [1.46346, 4.26703]** | n/a | 0.768750 [0.397676, 1.48607] | 1.02170 [0.611263, 1.70772] | ****0.318538 [0.185189, 0.547906]** | ****0.241659 [0.141897, 0.411559]** | ****6.49000E-02 [3.58806E-02, 0.117389]** | 2.14466 [0.496083, 9.27174] | ****0.387460 [0.241270, 0.62223**] | n/a | 2.22376 [0.895815, 5.52023] |
| Firewood | | ****0.101211 [4.75750E-02, 0.215316]** | ****0.120547 [5.64775E-02, 0.257297]** | **** 5.18640E-02 [1.52728E-02, 0.176122]** | ****0.161904 [9.23100E-02, 0.283967]** | n/a | ****0.201249 [8.85429E-02, 0.457417]** | ****4.45823E-02 [1.88055E-02, 0.105691]** | ****1.74150E-02 [7.74052E-03, 3.91811E-02]** | n/a | ****0.295150 [0.161487, 0.539444]** | n/a | ****0.209374 [0.104522, 0.419410]** | ****0.518137 [0.284105, 0.944955]** | ***3.108239 [1.532493, 6.304203]** | ***3.54671 [1.93187, 6.51141]** | ***20.0080 [9.22497, 43.3949** | ***0.125387 [2.82100E-02, 0.557333]** | 0.726544 [0.420692, 1.25476] | n/a | ****0.111318 [4.32500E-02, 0.286531]** |
| No mobile phone | | ****0.176139 [6.83960E-02, 0.453605]** | ****1.07994E-02 [3.82354E-03, 3.05023E-02]** | ****1.03774E-02 [1.02077E-03, 0.105500]** | **** 0.286024 [0.128134, 0.638471]** | n/a | ****3.88400E-02 [8.76000E-03, 0.172148]** | 0.520629 [0.201609, 1.344456] | 0.602019 [0.259556, 1.39633] | n/a | ****0.322986 [0.125287, 0.832646]** | n/a | ****5.42000E-03 [1.35000E-03, 2.18300E-02]** | ****0.110241 [4.35000E-02, 0.279390]** | ****0.175890 [7.0163E-02, 0.440938]** | 1.27604 [0.516640, 3.15164] | ***3.80156 [1.53415, 9.42010]** | ***6.18450E-04 [2.17999E-05, 1.75500E-02]** | ***8.53700E-02 [3.68300E-02, 0.197920]** | n/a | ****7.19751E-04 [7.81964E-05, 6.62000E-03]** |
| Absentee population | | 7.595306 [0.226787, 2.54374E+02] | ***72.918 [2.32527, 2.28663E+03]** | 3.12387E-03 [6.32717E-7, 15.4233] | ****1.79630E-02 [8.08116E-04, 0.399284]** | n/a | 7.23176E-03 [2.42164E-5, 2.15962] | ****2.15913E-02 [5.89674E-04, 0.790577]** | ****4.41189E-04 [1.43323E-5, 1.35800E-02]** | n/a | ****0.117882 [3.06381E-03, 4.53560]** | n/a | 7.19912 [0.120145, 4.31372E+02] | ***42.1032 [1.00751, 1.75946E+03]** | ***1.76076E+04 [4.64047E+03, 6.68095E+05]** | ***78.68443 [2.29036, 2.70318E+03]** | ***1.53271E+02 [4.53210, 5.18348E+03]** | 0.548679 [2.81826E-05, 1.06821E+04] | 2.61408 [0.138752, 49.2489] | n/a | 37.59254 [0.136043, 1.03879E+04] |
| Average number per household | | 1.181565 [0.950032, 1.469525] | 1.07612 [0.863498, 1.34109] | 1.089578 [0.653798, 1.815820] | ***1.66370 [1.37107, 2.01880]** | n/a | 0.884455 [0.631605, 1.23853] | ***1.79988 [1.37992, 2.34764]** | ***3.50177 [2.72455, 4.50071]** | n/a | 1.10459 [0.884299, 1.37975] | n/a | ****0.545473 [0.397849, 0.747873]** | ****0.703634 [0.566312, 0.874253]** | ****0.477534 [0.376790, 0.605215]** | ****0.642345 [0.515393, 0.800568]** | ****0.525503 [0.417334, 0.661709]** | 0.683279 [0.352855, 1.32312] | ****0.560719 [0.454135, 0.692318]** | n/a | ****0.371654 [0.223898, 0.616920]** |
| Indigenous population | | ****0.332592 [0.162835, 0.679320]** | 1.070071 [0.541925, 2.112933] | 0.213161 [3.95735E-02, 1.14818] | **** 0.124869 [6.36236E-02, 2.45070E-01]** | n/a | 0.350951 [0.114446, 1.07620] | ****0.146472 [6.74700E-02, 0.317983]** | ****4.47318E-02 [2.10646E-02, 9.49920E-02]** | n/a | 0.572249 [0.273634, 1.19674] | n/a | ****0.249513 [9.67000E-02, 0.643816]** | 0.55274 [0.274136, 1.11448] | 1.08706 [0.548590, 2.15405] | 1.57143 [0.786554, 3.13950] | 1.13772 [0.566036, 2.28679] | 1.01010 [0.132987, 7.67220] | ***2.91392 [1.59652, 5.31840]** | n/a | 1.15661 [0.341424, 3.91816] |
| MMR coverage | | ***4.65065 [1.74401, 12.4017]** | ***18.1431 [6.67551, 49.3102]** | ***17.213833 [2.361090, 1.25500E+02]** | 1.66964 [0.736849, 3.78326] | n/a | ***9.30010 [2.38328, 36.2911]** | 2.39642 [0.894600, 6.41943] | ***2.42212 [1.00616, 5.83078]** | n/a | 1.98203 [0.761116, 5.16144] | n/a | ***72.4843 [21.0679, 2.49382E+02]** | ***3.75770 [1.44323, 9.78385]** | ***5.949084 [2.278168, 15.535111]** | ***3.76245 [1.40532, 10.0732]** | ***23.9534 [8.70668, 65.8995]** | ****15.2910 [1.30807, 1.78748E+02]** | 0.647070 [0.285626, 1.46590] | n/a | ***25.3675 [5.17431, 1.24365E+02]** |
| Nutritional deficiency | | 6.22516E+07 [5.71827E-03, 6.77699E+17] | 2.98735 [6.38338E-11, 1.39804E+11] | 1.38997E+03 [1.43868E-19, 1.34292E+25] | 1.92001E+03 [4.05936E-6, 9.20242E+11] | n/a | 6.52168E-07 [9.47353E-24, 4.48959E+10] | 8.92885E-03 [7.28388E-14, 1.09453E+09] | 2.04276E+03 [1.11078E-6, 3.73220E+12] | n/a | 1.65890E+03 [1.38350E-7, 1.98840E+13] | n/a | 5.87554E+03 [7.51292E-09, 4.59501E+15] | 4.57759E-08 [2.85225E-19, 7.34659E+03] | 2.011562E+08 [3.9285E-02, 1.030E+18] | 10.7022 [5.28196E-10, 2.16844E+11] | 4.85365E+03 [4.21585E-07, 5.58795E+13] | 1.98380E+09 [8.28552E-17, 4.74982E+34] | 7.03247E+06 [1.89700E-02, 2.60742E+15] | n/a | 4.98389E-09 [1.03026E-29, 2.41096E+12] |
| Malnutrition | 2.23470E+25 [4.81536E-12, 1.03708E+62] | ***1.00780E+39 [6.34835E+03, 1.59988E+74]** | ***3.62429E+27 [1.95571E-54, 6.71645E+108]** | ***1.93109E+66, [1.31260E+35, 2.84099E+97]** | n/a | ***8.69478E+41 [5.41437E+10, 1.39627E+74]** | 3.94476E+28 [1.28323E-20, 1.21266E+77] | ***3.23229E+89 [1.11386E+50, 9.37972E+128]** | n/a | 1.08911E+24 [1.46572E-04, 8.09264E+51] | n/a | ***3.86257E+66 [8.08434E+36, 1.84547E+96]** | ***3.02063E+34 [22.5840, 4.04012E+67]** | 8.6024602E+09 [3.867E-27, 1.914E+46] | 1.31000E+12 [7.35485E-35, 2.33330E+58] | ***1.61265E+76 [5.32319E+35, 4.88552E+116]** | 1.89423E+36 [1.84388E-14, 1.94596E+86] | ****1.17426E-46 [2.45338E-83, 5.62032E-10]** | n/a | ***5.71552E+38 [1.31436E+02, 2.48540E+75]** |  |

**Bold text with *** indicates significant (p≤0.05) positive association (social determinant of health associated with more disease).

**Bold text with **** indicates significant (p>0.05) negative association (social determinant of health associated with less disease).

n/a: data did not distribute into a high, moderate and low category therefore only the high category was evaluated against the low category

Supplementary Table 3. Multivariable associations of social determinants of health with non-communicable disease outcomes (odds ratio [95% CI])

|  | Non-communicable diseases | | | | | | | | | | | |
| --- | --- | --- | --- | --- | --- | --- | --- | --- | --- | --- | --- | --- |
|  | Hypertension | | Diabetes | | COPD | | Liver Cirrhosis | | Depression | | Back pain | |
|  | Moderate vs low | High vs low | Moderate vs low | High vs low | Moderate vs low | High vs low | Moderate vs low | High vs low | Moderate vs low | High vs low | Moderate vs low | High vs low |
| Accessibility | REMOVED | REMOVED | ***1.87137 (1.41070 - 2.48249)** | ***3.09210 (2.20014 - 4.34569)** | 1.25103 (0.96179 - 1.62724) | ***1.68620 (1.26283 - 2.25151)** | REMOVED | REMOVED | ***1.48616 (1.18917 - 1.85733)** | ***2.40235 (1.86816 - 3.08928)** | REMOVED | REMOVED |
| Population under 5 | ****1.08116E-15 (2.90681E-22 - 4.02125E-09)** | ****2.07966E-15 (2.69916E-23 - 1.60235E-07)** | REMOVED | REMOVED | REMOVED | REMOVED | REMOVED | REMOVED | REMOVED | REMOVED | 1.83568 (0.00006 - 56458.55172) | ****4.96509E-09 (8.27613E-14 - 0.00030)** |
| Population over 65 | REMOVED | REMOVED | REMOVED | REMOVED | REMOVED | REMOVED | REMOVED | REMOVED | REMOVED | REMOVED | REMOVED | REMOVED |
| Illiteracy | REMOVED | REMOVED | REMOVED | REMOVED | 0.08037 (0.00483 - 1.33790) | 4.71999 (0.21828 - 102.06464) | REMOVED | REMOVED | REMOVED | REMOVED | 0.27543 (0.02249 - 3.37317) | ***43.30208 (3.19972 - 586.00966)** |
| Female proportion of population | ***5.91202E+08 (2293.96215 - 1.52365E+14)** | ***4.42114E+11 (370598.73451 - 5.27429E+17)** | REMOVED | REMOVED | ***1.71123E+06 (32.70014 - 8.95506E+10)** | ***1.76709E+07 (257.77940 - 1.21134E+12)** | ***2.68218E+07 (2189.09085 - 3.28635E+11)** | 130.94184 (0.02028 - 845288.75830) | REMOVED | REMOVED | REMOVED | REMOVED |
| Unemployment | REMOVED | REMOVED | REMOVED | REMOVED | ***1620.23733 (7.85786 - 334082.06761)** | ***9421.89273 (33.74487 - 2.63068E+06)** | REMOVED | REMOVED | REMOVED | REMOVED | REMOVED | REMOVED |
| No piped water | 0.54594 (0.22748 - 1.31021) | ****0.19030 (0.06789 - 0.53344)** | REMOVED | REMOVED | 0.54503 (0.23794 - 1.24842) | ****0.08240 (0.03197 - 0.21235)** | REMOVED | REMOVED | REMOVED | REMOVED | ****0.45007 (0.23551 - 0.86011)** | ****0.07532 (0.03660 - 0.15501)** |
| Firewood | ****0.26186 (0.09712 - 0.70600)** | ****0.13793 (0.04033 - 0.47178)** | 1.75284 (0.80136 - 3.83402) | ***3.53817 (1.30054 - 9.62572)** | REMOVED | REMOVED | REMOVED | REMOVED | ***4.29795 (1.97423 - 9.35676)** | ***13.12635 (5.21766 - 33.02267)** | REMOVED | REMOVED |
| No mobile phone | 0.52154 (0.11070 - 2.45715) | ****0.05277 (0.00889 - 0.31313)** | ****0.17622 (0.04115 - 0.75469)** | ****0.00229 (0.00034 - 0.01565)** | REMOVED | REMOVED | REMOVED | REMOVED | REMOVED | REMOVED | REMOVED | REMOVED |
| Absentee population | REMOVED | REMOVED | REMOVED | REMOVED | REMOVED | REMOVED | REMOVED | REMOVED | REMOVED | REMOVED | REMOVED | REMOVED |
| Average number per household | 0.99313 (0.64067 - 1.53948) | ****0.36978 (0.20711 - 0.66022)** | ****0.73206 (0.55163 - 0.97151)** | ****0.48376 (0.33306 - 0.70262)** | 0.84656 (0.58572 - 1.22357) | ****0.36566 (0.23563 - 0.56744)** | ***1.54008 (1.12989 - 2.09918)** | 0.78595 (0.58305 - 1.05947) | ****0.76029 (0.58160 - 0.99388)** | ****0.30854 (0.22375 - 0.42545)** | REMOVED | REMOVED |
| Indigenous population | REMOVED | REMOVED | REMOVED | REMOVED | 0.49891 (0.21049 - 1.18254) | ****0.10027 (0.03649 - 0.27555)** | 1.49658 (0.68157 - 3.28620) | 0.51485 (0.22206 - 1.19373) | 0.75675 (0.33878 - 1.69039) | ****0.14174 (0.05426 - 0.37029)** | 0.64277 (0.27061 - 1.52678) | ****0.22586 (0.08914 - 0.57225)** |
| MMR coverage | REMOVED | REMOVED | 0.88862 (0.30273 - 2.60837) | ***3.80536 (1.02701 - 14.09994)** | ***3.21530 (1.04474 - 9.89546)** | ***6.26858 (1.87505 - 20.95677)** | REMOVED | REMOVED | REMOVED | REMOVED | 2.16510 (0.77519 - 6.04710) | ***6.14476 (2.13575 - 17.67908)** |
| Nutritional deficiency | 97.14666 (7.14181E-13 - 1.32144E+16) | ***6.10887E+16 (48.10284 - 7.75802E+31)** | REMOVED | REMOVED | REMOVED | REMOVED | REMOVED | REMOVED | REMOVED | REMOVED | ***3.84486E+15 (50.53292 - 2.92541E+29)** | ***2.50950E+37 (7.09619E+23 - 8.87462E+50)** |
| Malnutrition | ***1.60696E+43 (139.32496 - 1.85345E+84)** | ***5.13021E+91 (4.44456E+44 - 5.92163E+138)** | 3.21662E+32 (4.72508E-10 - 2.18973E+74) | ***1.60157E+89 (1.34349E+42 - 1.90924E+136)** | ***4.11973E+68 (1.34310E+10 - 1.26365E+127)** | ***6.10532E+163 (7.55248E+102 - 4.93545E+224)** | 1.70292E+30 (9.39843E-23 - 3.08554E+82) | ***9.45492E+95 (1.04925E+49 - 8.51996E+142)** | ***1.79493E+61 (5.47359E+12 - 5.88600E+109)** | ***3.24583E+104 (1.97692E+54 - 5.32922E+154)** | REMOVED | REMOVED |

**Bold text with *** indicates significant (p≤0.05) positive association (social determinant of health associated with more disease).

**Bold text with **** indicates significant (p>0.05) negative association (social determinant of health associated with less disease).

Removed: Variable removed by model in backwards selection multivariable analysis

Supplementary Table 4. Multivariable associations of social determinants of health with infectious disease outcomes (odds ratio [95% CI])

|  | Infectious diseases | | | | | | | | | | | | | | | | | | | |
| --- | --- | --- | --- | --- | --- | --- | --- | --- | --- | --- | --- | --- | --- | --- | --- | --- | --- | --- | --- | --- |
|  | TB | | Malaria | | Kala azar | | Leprosy | | Filariasis | | HIV | | Influenza | | Pneumonia | | Measles | | Dengue | |
|  | Moderate vs low | High vs low | Moderate vs low | High vs low | Moderate vs low | High vs low | Moderate vs low | High vs low | Moderate vs low | High vs low | Moderate vs low | High vs low | Moderate vs low | High vs low | Moderate vs low | High vs low | Moderate vs low | High vs low | Moderate vs low | High vs low |
| Accessibility | ***3.22462 [2.29438 - 4.53200]** | ***5.70091 [3.86986 - 8.39833]** | ***4.87362 [2.55465 - 9.29761]** | ***1.80662 [1.48127 - 2.20344]** | n/a | ***2.45305 [1.76388 - 3.41150]** | ***1.67886 [1.21266 - 2.32429]** | ***1.91920 [1.33827 - 2.75233]** | n/a | ***1.53655 [1.26166- 1.87132]** | n/a | ***4.67149 [3.29321 - 6.62662]** | ***1.71694 [1.33787 - 2.20341]** | ***1.26650 [.99084 - 1.61886]** | REMOVED | REMOVED | ***9.70679 [2.69777 - 34.92583]** | ***1.56904 [1.23537 - 1.99283]** | n/a | ***2.53165 [1.40998 - 4.54562]** |
| Population under 5 | 691588.841544 [.37599 - 1.27209E+12] | ***1.74250E+19 [3.61069E+11 - 8.40925E+26]** | REMOVED | REMOVED | n/a | REMOVED | ***7.118E+10[29028.37323 - 1.74547E+17]** | 1.566875E+6 [.16871 - 1.46305E+13] | n/a | REMOVED | n/a | REMOVED | REMOVED | REMOVED | ***9.812646E+6 [25.26039 - 3.75825E+12]** | ***516030000000000 [455411330 - 5.847E+20]** | 1.38817E-21 [3.34130E-45 - 576.72194] | ****3.94028E-12 [1.44914E-16 - 1.07138E-7]** | n/a | REMOVED |
| Population over 65 | REMOVED | REMOVED | REMOVED | REMOVED | n/a | ****4.9177E-11 [3.1485E-17 - 7.68080E-5]** | REMOVED | REMOVED | n/a | REMOVED | n/a | REMOVED | 0.00343 [2.85919E-6 - 4.11814] | 25.01148 [.10226 - 6117.43199] | REMOVED | REMOVED | ****5.7048E-14 [4.47085E-26 - 0.07279]** | 0.00899 [2.70364E-5 - 2.99151] | n/a | REMOVED |
| Illiteracy | REMOVED | REMOVED | REMOVED | REMOVED | n/a | REMOVED | ****0.006024 [0.00016 - 0.23361]** | 0.20971 [.00602 - 7.95792] | n/a | REMOVED | n/a | REMOVED | 1.04948 [.07216 - 15.26423] | ****0.02770 [0.00166 - 0.46217]** | REMOVED | REMOVED | 9071.41298 [.53308 - 1.54368E+8] | ***70.62139 [6.74570 - 739.34210]** | n/a | ****0.00107 [7.07062E-6 - 0.16313]** |
| Female proportion of population | 75728.68579 [.007 - 8.544E+11] | ****2.3048E-11 [2.2595E-19 - 0.00242]** | REMOVED | REMOVED | n/a | ****2.64042E-9 [2.50005E-15 - 0.00279]** | 9.51318E+6 [.02157 - 4.19598E+15] | 5.93468E-6[1.37489E-14 - 2561.69980] | n/a | ****4.07460E-5 [1.97842E-8 - 0.08392]** | n/a | REMOVED | REMOVED | REMOVED | REMOVED | REMOVED | REMOVED | REMOVED | n/a | REMOVED |
| Unemployment | REMOVED | REMOVED | REMOVED | REMOVED | n/a | REMOVED | REMOVED | REMOVED | n/a | REMOVED | n/a | REMOVED | REMOVED | REMOVED | REMOVED | REMOVED | REMOVED | REMOVED | n/a | ****5.7613E-12 [6.15618E-22 - .05392]** |
| No piped water | ***2.96489 [1.17571 - 7.47686]** | ***12.35807 [4.71628 - 32.38184]** | ***19.52203 [4.22607 - 90.18068]** | ***6.04971 [3.46543 - 10.56116]** | n/a | REMOVED | ***20.39056 [7.05651 - 58.92081]** | ***107.87875 [34.98857 - 332.61787]** | n/a | REMOVED | n/a | ****0.16887 [0.07011 - 0.40679]** | REMOVED | REMOVED | ****0.38312 [0.17754 - 0.82675]** | ****0.13611 [0.05464 - 0.33906]** | 0.29412 [.03183 - 2.71755] | ****0.18561 [0.09447 - 0.36227]** | n/a | REMOVED |
| Firewood | 0.476443 [.14980 - 1.51530] | 3.51496 [.96782 - 12.76581] | REMOVED | REMOVED | n/a | REMOVED | ****0.20361 [0.05681 - 0.72977]** | 0.481502 [.13549 - 1.71115] | n/a | REMOVED | n/a | REMOVED | 0.94862 [.41134 - 2.18814] | ***3.77415 [1.45323 - 9.80177]** | 1.58087 [.71554 - 3.49264] | ***4.29391 [1.48863 - 12.38584]** | REMOVED | REMOVED | n/a | REMOVED |
| No mobile phone | 2.74334 [.47445 - 15.86245] | ****0.04285 [0.00512 - 0.35865]** | REMOVED | REMOVED | n/a | REMOVED | REMOVED | REMOVED | n/a | REMOVED | n/a | REMOVED | REMOVED | REMOVED | REMOVED | REMOVED | REMOVED | REMOVED | n/a | REMOVED |
| Absentee population | 4.77819 [.00517 - 4412.41755] | ***632027.58698 [221.82008 - 1.8008E+9]** | REMOVED | REMOVED | n/a | REMOVED | 0.00248 [7.09595E-7 - 8.69882 | ***6283.33007 [2.15787 - 1.82959E+7]** | n/a | REMOVED | n/a | REMOVED | REMOVED | REMOVED | 0.09212 [.00096 - 8.81721] | ****0.00199 [1.53618E-5 - 0.25770]** | REMOVED | REMOVED | n/a | REMOVED |
| Average number per household | REMOVED | REMOVED | REMOVED | REMOVED | n/a | REMOVED | 1.35696 [.75993 - 2.42303] | ***2.15376 [1.19513 - 3.88131]** | n/a | REMOVED | n/a | REMOVED | ****0.55079 [0.39465 - 0.76872]** | ****0.54447 [0.38367 - 0.77267]** | ****0.56163 [0.36928 - 0.85418]** | ****0.27051 [0.16006 - 0.45719]** | REMOVED | REMOVED | n/a | REMOVED |
| Indigenous population | 0.98636 [.35796 - 2.71790] | ***11.97738 [3.74838 - 38.27190]** | 0.14408 [.01209 - 1.71718] | ****0.16601 [0.07361 - 0.37441]** | n/a | REMOVED | REMOVED | REMOVED | n/a | REMOVED | n/a | ****0.04798 [0.00962 - 0.23937]** | ****0.30531 [0.12608 - 0.73931]** | ****0.25364 [0.10775 - 0.59710]** | REMOVED | REMOVED | REMOVED | REMOVED | n/a | REMOVED |
| MMR coverage | ***9.86051 [3.03878 - 31.99633]** | ***60.70938 [16.12268 - 228.59906]** | ***23.91956 [2.20532 - 259.43852]** | ***2.81929 [1.11064 - 7.15656]** | n/a | ***9.50830 [2.03096 - 44.51481]** | ***4.25197 [1.26693 - 14.27010]** | ***30.59976 [8.57610 - 109.18076]** | n/a | REMOVED | n/a | ***34.51993 [8.23674 - 144.67200]** | REMOVED | REMOVED | 2.34680 [.81209 - 6.78184] | ***10.26862 [3.17030 - 33.26009]** | REMOVED | REMOVED | n/a | ***7.27056 [1.17364 - 45.04024]** |
| Nutritional deficiency | REMOVED | REMOVED | REMOVED | REMOVED | n/a | REMOVED | REMOVED | REMOVED | n/a | REMOVED | n/a | REMOVED | ****0.00000 [6.67428E-27 - 0.14924]** | 5323838.302681 [5.298E-5 - 5.350E+17] | REMOVED | REMOVED | REMOVED | REMOVED | n/a | REMOVED |
| Malnutrition | REMOVED | REMOVED | REMOVED | REMOVED | n/a | REMOVED | REMOVED | REMOVED | n/a | REMOVED | n/a | ***2.70342E+75 [1.23263E+34 - 5.929E+116]** | ***5.5508E+52 [1.498E+12 - 2.057E+93]** | 1.4594E+39 [.000 - 6.399E+81] | ***4.01013E+75 [4.62363E+15 - 3.47803E+135]** | ***1.87532E+168 [4.87730E+104 - 7.21057E+231]** | REMOVED | REMOVED | n/a | ***1.61271E+49 [32.06563 - 8.11092E+96]** |

**Bold text with *** indicates significant (p≤0.05) positive association (social determinant of health associated with more disease).

**Bold text with **** indicates significant (p>0.05) negative association (social determinant of health associated with less disease).

Removed: Variable removed by model in backwards selection multivariable analysis

n/a: data did not distribute into a high, moderate and low category therefore only the high category was evaluated against the low category
